# Enhancing the Solubility of Isoconazole Nitrate Using Methyl-β-Cyclodextrin: Formulation and Characterization of Inclusion Complexes

**DOI:** 10.3390/molecules30081654

**Published:** 2025-04-08

**Authors:** Tarek Alloush, Gülsel Yurtdaş Kırımlıoğlu

**Affiliations:** 1 Department of Pharmaceutical Technology, Institute of Health Sciences, Istanbul University, Istanbul 34126, Türkiye; 2 Department of Pharmaceutical Technology, Graduate School of Health Sciences, Anadolu University, Eskişehir 26470, Türkiye; 3 Department of Pharmaceutical Technology, Faculty of Pharmacy, Anadolu University, Eskişehir 26470, Türkiye; gyurtdas@anadolu.edu.tr

**Keywords:** isoconazole nitrate, methyl-β-cyclodextrin, spray-drying, freeze-drying, inclusion complex, phase solubility

## Abstract

Isoconazole nitrate (ISN) is a broad-spectrum antifungal agent whose therapeutic potential is limited by poor aqueous solubility and low bioavailability. This study aimed to enhance the solubility and physicochemical properties of ISN through the formation of inclusion complexes with methyl-β-cyclodextrin (M-β-CD) using freeze-drying (FD) and spray-drying (SD) methods. The prepared inclusion complexes were comprehensively characterized by high-performance liquid chromatography (HPLC), phase solubility analysis, scanning electron microscopy (SEM), differential scanning calorimetry (DSC), Fourier-transform infrared spectroscopy (FT-IR), and proton nuclear magnetic resonance (^1^H-NMR). Phase solubility studies revealed an AL-type solubility diagram with a 1:1 molar ratio and an apparent stability constant (*K_S_*) of 2711 M^−1^. Structural and thermal analyses confirmed successful inclusion complex formation and reduced crystallinity. The solubility assessment showed that ISN/M-β-CD complexes prepared by SD exhibited an approximately seven-fold higher aqueous solubility than ISN and performed better than those prepared by FD. Moreover, SD complexes demonstrated a higher drug content. These findings highlight the potential of M-β-CD-based inclusion complexation, particularly via spray-drying, as an effective strategy to enhance the solubility and bioavailability of poorly water-soluble drugs, such as ISN.

## 1. Introduction

Isoconazole nitrate (ISN) is a broad-spectrum azole-class antifungal agent with strong activity against dermatophytes, yeasts, molds, and Gram-positive bacteria [1]. Its primary antifungal mechanism involves the inhibition of 14α-demethylase, a crucial enzyme in the ergosterol biosynthesis pathway in fungi [2]. This inhibition disrupts the conversion of lanosterol to ergosterol, leading to an accumulation of toxic sterol precursors, such as 14α-methylsterols [3]. These abnormal sterol intermediates impair membrane integrity, increase permeability, and ultimately cause fungal cell death [4]. Owing to this targeted mode of action, ISN exhibits both fungistatic and fungicidal properties, making it highly effective for treating cutaneous and mucosal fungal infections [5].

Despite its therapeutic benefits, ISN, as an active pharmaceutical ingredient (API), exhibits poor aqueous solubility (0.5088 ± 0.0062 mg/mL in water) and light sensitivity, which significantly limit its bioavailability and clinical effectiveness [5]. According to the Biopharmaceutics Classification System (BCS), ISN is a Class II drug, characterized by low solubility but high permeability [6]. Enhancing drug solubility is a major pharmaceutical challenge, and multiple formulation approaches have been investigated, including particle size reduction, lipid-based delivery systems, and inclusion complexation [7,8].

Among these strategies, cyclodextrins (CDs) have been widely studied due to their ability to enhance the solubility, stability, and bioavailability of poorly water-soluble drugs [8,9]. Cyclodextrins (CDs) are cyclic oligosaccharides composed of α-(1,4)-linked glucopyranose units, characterized by a hydrophobic internal cavity and a hydrophilic exterior. This structural feature enables them to incorporate hydrophobic drug molecules through non-covalent interactions, resulting in the formation of inclusion complexes that enhance drug solubility and stability [10,11,12]. The commonly used natural CDs—α-CD, β-CD, and γ-CD—differ in cavity diameter, which determines their ability to accommodate various guest molecules [13,14]. Among their derivatives, methyl-β-cyclodextrin (M-β-CD) exhibits superior aqueous solubility and higher complexation efficiency, making it a favorable carrier for poorly soluble drugs [15,16].

In addition to cyclodextrin-based inclusion complexation, recent research has also emphasized the potential of peptide–drug conjugation as a promising strategy to improve drug solubility and pharmacological performance. This approach involves the covalent attachment of peptides to small-molecule drugs, which can significantly enhance their aqueous solubility and delivery efficiency. For instance, Fu et al. reported that peptide conjugation increased the solubility of chemotherapeutic agents by nearly 700-fold, offering compelling evidence for the efficacy of this strategy in overcoming solubility limitations and improving therapeutic outcomes [17]. Although this strategy differs mechanistically from inclusion complexation, it highlights the significance of innovative formulation techniques in addressing solubility challenges in pharmaceutical development [18,19,20].

**Figure 1:**
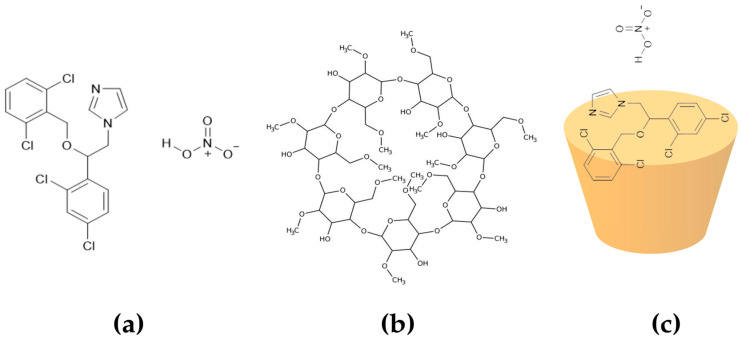
Chemical structures of (**a**) isoconazole nitrate and (**b**) methyl-β-cyclodextrin and (**c**) the schematic representation of the inclusion complex formation mechanism (ISN/M-β-CD).

To illustrate the structural characteristics of ISN, M-β-CD, and their complex formation process, Figure 1 presents their chemical structures, along with a schematic representation of the inclusion complexation mechanism.

The aim of this study is to develop and characterize ISN/M-β-CD inclusion complexes using freeze-drying (FD) and spray-drying (SD) methods to enhance the physicochemical and biopharmaceutical properties of ISN. The formation of inclusion complexes with M-β-CD is expected to increase ISN solubility, improve its dissolution rate, and enhance photostability, ultimately leading to improved therapeutic performance. Additionally, this study explores how different complexation techniques influence molecular interactions, drug release profiles, and solid-state properties.

## 2. Results and Discussion

### 2.1. Determination of Purity and Quantification of Isoconazole Nitrate via HPLC

**Figure 2:**
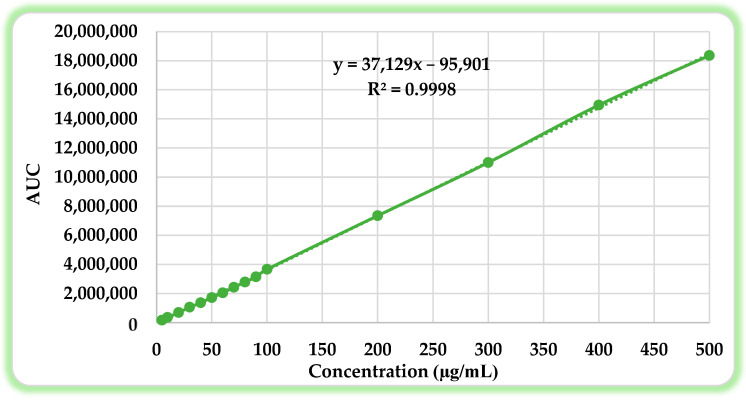
Calibration curve for ISN quantification and the linearity equation using the validated HPLC method [8].

The quantification and purity analysis of isoconazole nitrate (ISN) were performed using a validated high-performance liquid chromatography (HPLC) method in accordance with standard analytical practices. The method demonstrated excellent linearity, with a calibration curve described by the regression equation: y = 37,129x − 95,901 and a correlation coefficient (R^2^ = 0.9998), indicating a strong relationship between the concentration and the peak area (Figure 2). The method’s accuracy was verified through recovery tests, with values ranging from 99.24% to 101.19%, which fell within the acceptable recovery range of 98–102% [21].

Precision was assessed at four concentration levels, and the relative standard deviation (RSD%) values remained below 2%, confirming the high reproducibility [22]. The method’s sensitivity was further demonstrated by its limit of detection (LOD) and limit of quantification (LOQ), calculated to be 1.30 μg/mL and 3.95 μg/mL, respectively. Furthermore, the method exhibited strong selectivity, with ISN peaks clearly resolved under the chromatographic conditions and no interference observed from excipients or solvent components. The results obtained collectively affirmed the reliability, accuracy, precision, and selectivity of the developed HPLC method for the analysis of ISN [23].

### 2.2. Characterization of ISN/M-β-CD Inclusion Complexes

#### 2.2.1. Phase Solubility Studies and Stability Analysis

Phase solubility analysis is a fundamental approach used to assess both the solubilizing capability and the stability of inclusion complexes, especially for drugs with limited water solubility [24]. The solubility profile is the basis for the subsequent categorization of diagrams derived from this analysis. Two primary categories are generally recognized: A-type (including AL, AN, and AP) and B-type (BI and BS). A-type curves typically indicate the formation of soluble inclusion complexes, while B-type profiles reflect limited solubility due to the precipitation of the complex [25]. While natural cyclodextrins (CDs) often produce B-type solubility curves due to their limited water solubility, chemically modified CDs, such as methyl-β-cyclodextrin (M-β-CD), can generate soluble inclusion complexes.

To determine the molar ratio between ISN and M-β-CD, a phase solubility study was conducted using M-β-CD solutions (20 mM) with an excess amount of ISN. The system was allowed to reach equilibrium over three days, with 24 h confirmed as the equilibrium time for M-β-CD solutions. The phase solubility diagram (Figure 2) was obtained by plotting the solubilized ISN concentration against the M-β-CD concentration, confirming that the system followed an AL-type solubility profile, which is indicative of 1:1 complexation [24]. The slope of the solubility curve (0.5866) remained below 1, further verifying the 1:1 host–guest inclusion complex formation [26].

The apparent stability constant (*K_S_*) for the M-β-CD complex was determined using the Higuchi and Connors equation [27]:

*K_S_* = *slope*/[*intercept*(1 − *slope*)]

**Figure 3:**
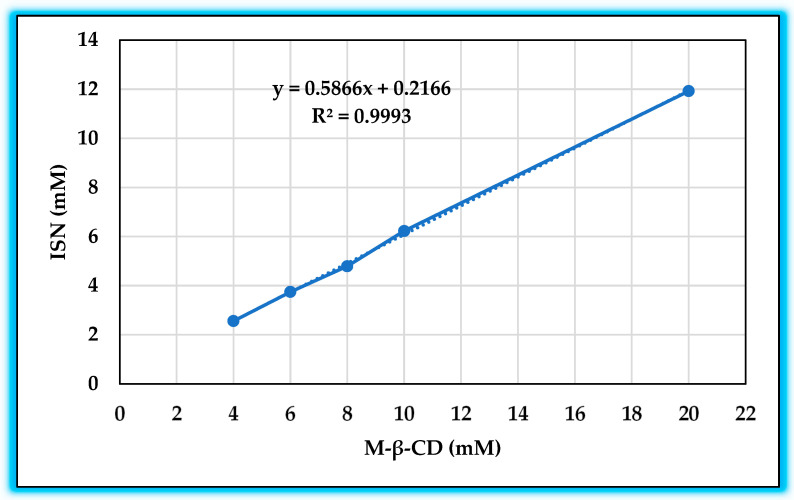
Phase solubility diagram of ISN/M-β-CDs.

Based on this equation, the calculated stability constant (*K_S_*) for the ISN/M-β-CD complex was 2711 M^−1^. The calculated complexation efficiency (*CE*) was 1.417, reflecting the complex’s strong drug-loading capability. The accuracy of the solubility curve could be further improved by removing the first datapoint in Figure 3, as it appeared to be an outlier that slightly skewed the regression analysis.

The presence of ethanol in the phase solubility system was intended to enhance the solubility of ISN, considering its low aqueous solubility. However, binding constants obtained in ethanol–water mixtures should not be directly compared to those in pure aqueous systems, as ethanol influences complexation efficiency and solubilization behavior. Future studies should focus on determining binding constants in an entirely aqueous system for a more precise evaluation of complex stability. Additionally, complexation efficiency (*CE*), which can be calculated as *CE* = *slope* / (1 − *slope*), provides a critical measure of drug-loading capacity and should be reported to further assess the pharmaceutical relevance of the inclusion complex.

#### 2.2.2. Drug Content (DC) Determination

The DC% of ISN incorporated into the M-β-CD inclusion complexes was quantified to assess the efficiency of drug encapsulation. The results indicated that the freeze-drying method yielded an ISN content of 23.2% ± 1.4%, while the spray-drying method resulted in a slightly higher drug loading of 24.0% ± 0.2%. These values demonstrated the efficient incorporation of ISN into the M-β-CD complex, with minimal variation between the two preparation techniques.

#### 2.2.3. Solubility Assessment of ISN/M-β-CD Inclusion Complexes

The solubility of ISN/M-β-CD inclusion complexes was evaluated at room temperature (25 °C) in distilled water, under ambient humidity conditions. It has been well established that cyclodextrin (CD) molecules enhance the solubility of poorly water-soluble guest compounds through inclusion complexation [28,29].

The saturated aqueous solubility of ISN was determined to be 0.5088 ± 0.0062 mg/mL. However, the formation of ISN/M-β-CD inclusion complexes significantly enhanced its solubility. The freeze-dried (FD) complex exhibited a solubility of 2.5684 ± 0.1636 mg/mL, whereas the spray-dried (SD) complex demonstrated an even greater improvement, reaching 3.8869 ± 0.5084 mg/mL. These results indicated that spray-drying led to superior solubility enhancement compared to freeze-drying. The higher solubility observed in SD-prepared complexes may be attributed to their smaller particle size and narrower particle size distribution, leading to an increased surface area for dissolution.

Furthermore, the solubility of ISN/M-β-CD complexes was found to be approximately seven times higher than that of ISN. The remarkable improvement in saturated aqueous solubility observed for both FD and SD complexes can be attributed to inclusion complexation, which promotes an amorphous state and reduces post-complexation crystallinity, thereby facilitating dissolution [29,30,31].

Additionally, the ISN/M-β-CD spray-dried complex in this study (3.8869 mg/mL) demonstrated slightly higher solubility compared to the ISN/hydroxypropyl-β-cyclodextrin (HP-β-CD) spray-dried complex previously reported in literature (3.6550 mg/mL) [8]. This suggests that M-β-CD is an effective carrier for enhancing the aqueous solubility of ISN, offering comparable or superior performance to HP-β-CD.

#### 2.2.4. Morphological Evaluation via Scanning Electron Microscopy (SEM)

**Figure 4:**
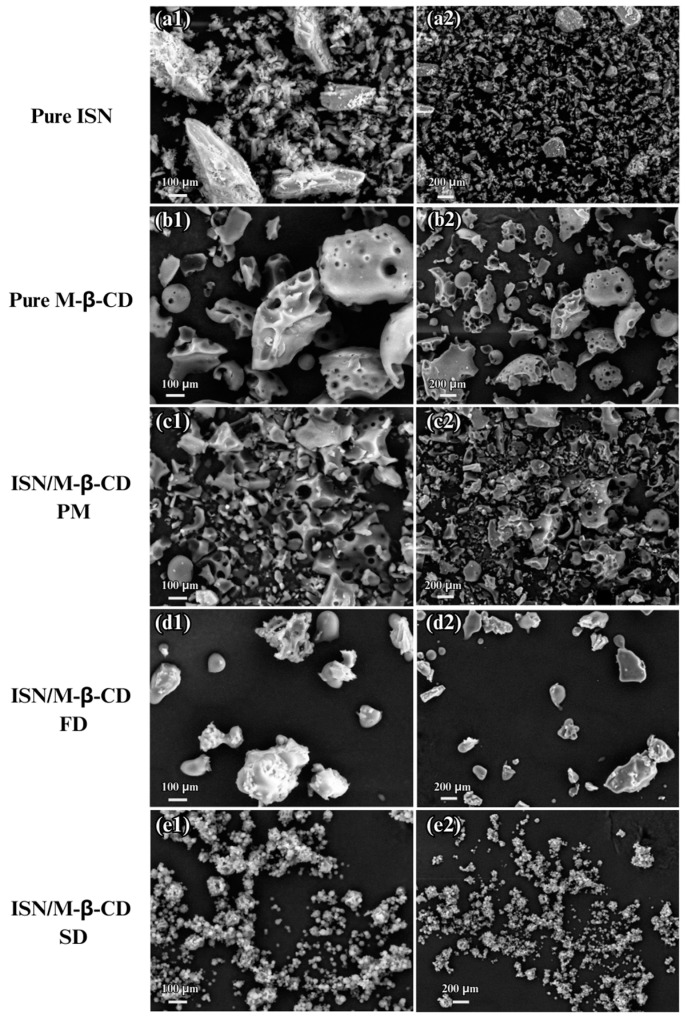
SEM images of ISN (**a1**,**a2**), M-β-CD (**b1**,**b2**), ISN/M-β-CD physical mixture (**c1**,**c2**), ISN/M-β-CD freeze-dried inclusion complex (**d1**,**d2**), and ISN/M-β-CD spray-dried inclusion complex (**e1**,**e2**).

The morphological characteristics of ISN, M-β-CD, their physical mixture (PM), and ISN/M-β-CD inclusion complexes prepared via freeze-drying (FD) and spray-drying (SD) were analyzed using scanning electron microscopy (Figure 4). The SEM images of ISN exhibited irregularly shaped crystalline particles, indicative of its semi-crystalline nature, while M-β-CD appeared as amorphous, porous particles, facilitating drug entrapment and inclusion complexation. The PM sample retained the distinct morphologies of both components, suggesting a lack of molecular interaction.

A comparison of FD and SD complexes revealed significant morphological differences. The FD complex formed larger, aggregated particles with a porous structure, indicating partial crystallinity retention. In contrast, the SD complex presented smaller, homogeneously dispersed amorphous particles, suggesting a greater disruption of ISN’s crystalline form and enhanced molecular dispersion. The finer particle size and increased surface area of the SD complex contribute to its superior solubility and dissolution rate, as confirmed in solubility studies. These findings indicate that spray-drying is a more effective method for enhancing the solubility and bioavailability of ISN through inclusion complexation with M-β-CD [30,32].

#### 2.2.5. Thermal Behavior Analysis via Differential Scanning Calorimetry (DSC)

**Figure 5:**
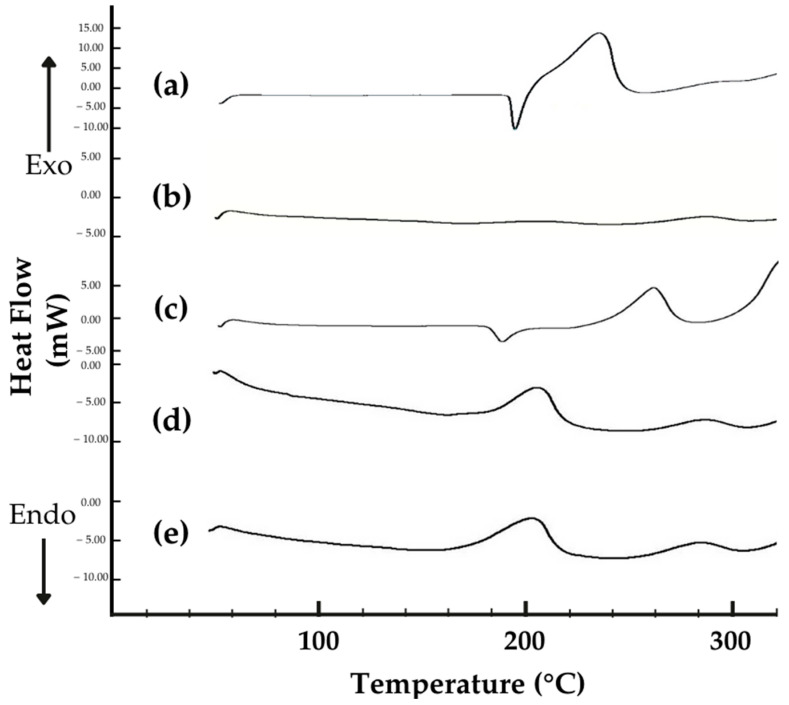
DSC thermograms of (**a**) ISN, (**b**) M-β-CD, (**c**) ISN/M-β-CD physical mixture, (**d**) ISN/M-β-CD freeze-dried complex, and (**e**) ISN/M-β-CD spray-dried complex.

The DSC thermograms of ISN, M-β-CD, their physical mixture (PM), and the ISN/M-β-CD inclusion complexes prepared by freeze-drying (FD) and spray-drying (SD) were analyzed over a temperature range of 50–300 °C at a heating rate of 10 °C/min. Approximately 4 mg of each sample was sealed in an aluminum pan for analysis. The DSC thermograms (Figure 5) provided insights into phase transitions, crystallinity changes, and complex formation [33,34].

The DSC thermogram of ISN (Figure 5a) exhibited a sharp endothermic melting peak at 187.5 °C, confirming its crystalline nature. In contrast, the M-β-CD thermogram (Figure 5b) displayed a broad endothermic event, attributed to water loss [32,35].

The physical mixture (Figure 5c) retained a less intense ISN melting peak, indicating a partial retention of crystalline ISN and the absence of full complexation. This suggests a limited interaction between ISN and M-β-CD in the solid state.

The FD (Figure 5d) and SD (Figure 5e) inclusion complexes showed a complete disappearance of the ISN melting peak, confirming successful drug encapsulation and amorphization. The broader thermal events in these complexes indicated stronger host–guest interactions, leading to enhanced physicochemical stability [36,37]. A broad exothermic anomaly between 200–210 °C was observed in both FD and SD samples, which likely corresponded to structural rearrangements or recrystallization phenomena within the cyclodextrin matrix.

#### 2.2.6. Functional Group Interactions via Fourier-Transform Infrared Spectroscopy (FT-IR)

**Figure 6:**
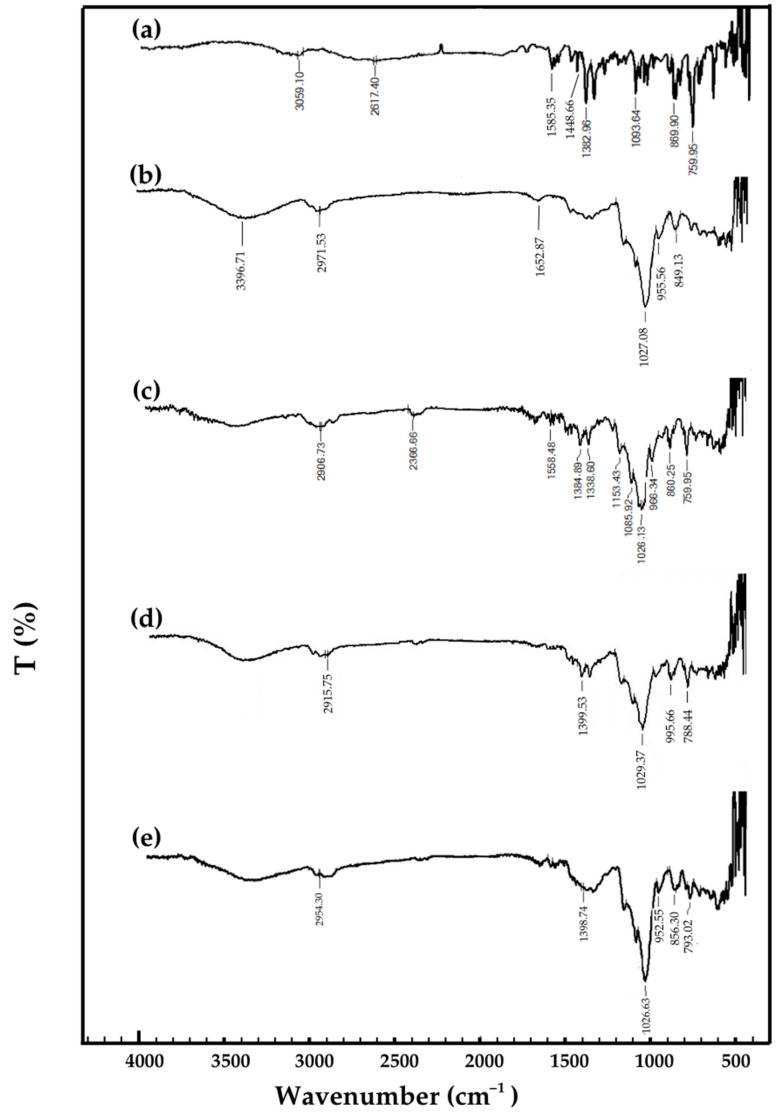
FT-IR spectra of (**a**) ISN, (**b**) M-β-CD, (**c**) ISN/M-β-CD physical mixture, (**d**) ISN/M-β-CD freeze-dried complex, and (**e**) ISN/M-β-CD spray-dried complex.

FT-IR was employed to investigate molecular interactions between ISN and M-β-CD during inclusion complex formation. The FT-IR spectra (Figure 6) provided insights into changes in characteristic functional groups, confirming host–guest interactions within the complexes.

The ISN spectrum (Figure 6a) exhibited distinct bands at 3059 cm^−1^ (aromatic C-H stretching), 1585 cm^−1^ (C = C stretching of the aromatic ring), 1448 cm^−1^ (C = N stretching), 1093 cm^−1^ (C-O-C stretching), and 759 cm^−1^ (C-Cl stretching), which align with previously reported ISN spectra [8,38]. The M-β-CD spectrum (Figure 6b) displayed broad bands at 3397 cm^−1^ (O-H stretching, indicative of hydrogen bonding), 2972 cm^−1^ (C-H stretching), and 1653 cm^−1^ (H–O–H bending of adsorbed water molecules). Additionally, a strong band at 1027 cm^−1^ was assigned to C–O–C and C–O stretching vibrations, confirming the glucopyranose ring structure typical of cyclodextrin derivatives [33,39].

The physical mixture (PM) spectrum (Figure 6c) retained characteristic peaks of both ISN and M-β-CD without significant peak shifts or intensity reductions, suggesting that no strong molecular interactions occurred in the physical blend. However, in the freeze-dried (FD) (Figure 6d) and spray-dried (SD) (Figure 6e) complexes, notable shifts and intensity reductions were observed in key ISN absorption bands, particularly at 1585 cm^−1^ (C = C stretching) and 1449 cm^−1^ (C = N stretching). These spectral modifications indicate intermolecular interactions, likely involving hydrogen bonding and the encapsulation of ISN within the M-β-CD cavity.

A shift in the C-Cl stretching band to higher wavenumbers was observed in both FD and SD inclusion complexes, further supporting host–guest interactions within the cyclodextrin cavity. The disappearance or weakening of ISN-specific peaks in the FD and SD spectra suggests that the drug was successfully incorporated into the cyclodextrin matrix, confirming the formation of stable inclusion complexes. These spectral changes align with findings from DSC and solubility studies, reinforcing the enhanced physicochemical properties of ISN when complexed with M-β-CD.

#### 2.2.7. Structural Confirmation via Proton Nuclear Magnetic Resonance (¹H-NMR) Analysis

^1^H-NMR spectroscopy was employed to confirm the formation of ISN/M-β-CD inclusion complexes by examining chemical shift variations (Δδ) in the proton environments of M-β-CD. When a guest molecule is incorporated into the cyclodextrin cavity, non-covalent interactions, such as van der Waals forces, hydrogen bonding, and hydrophobic interactions, can alter the chemical environment of the cyclodextrin’s internal protons, particularly H-3 and H-5, leading to observable shifts in the ¹H-NMR spectrum.

**Figure 7:**
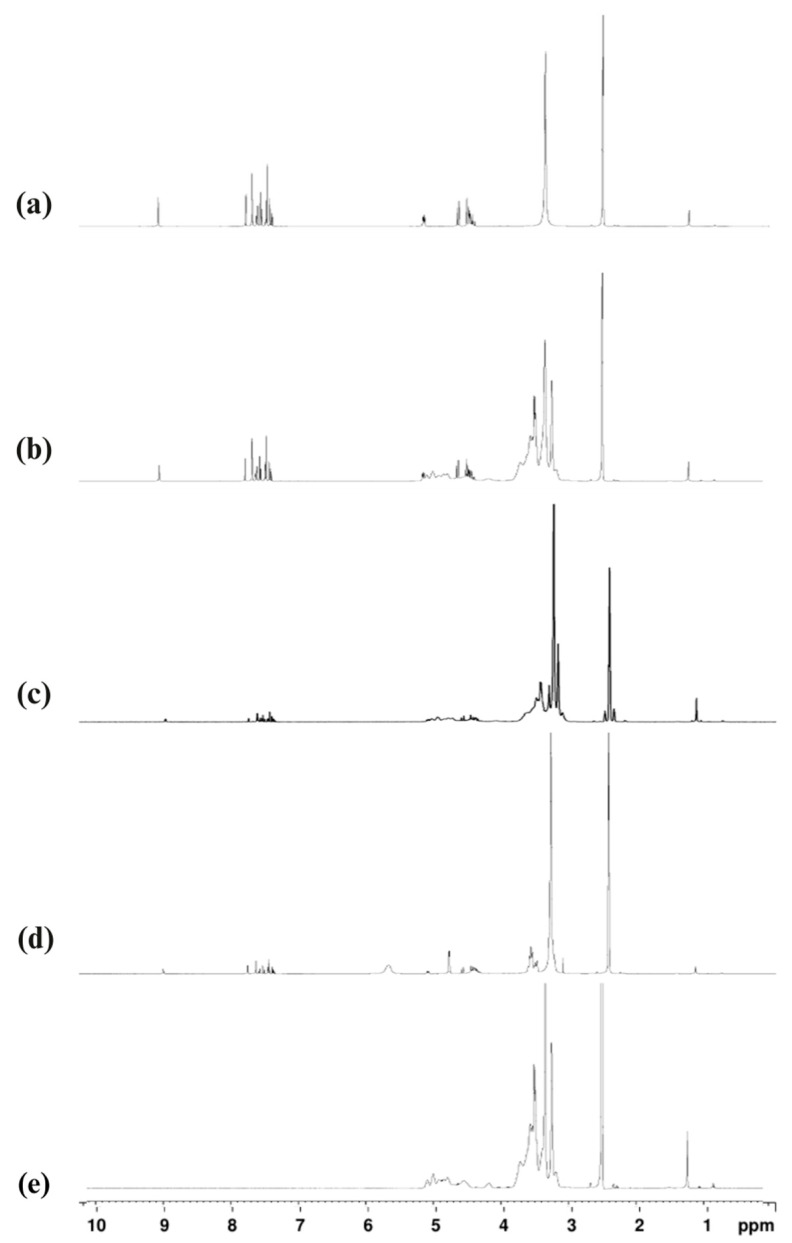
^1^H-NMR spectra of (**a**) ISN, (**b**) M-β-CD, (**c**) ISN/M-β-CD PM, (**d**) ISN/M-β-CD FD, and (**e**) ISN/M-β-CD SD.

The spectrum of ISN (Figure 7a) displayed characteristic signals at 8.79 ppm (singlet, N^+^–H), 7.16–7.55 ppm (multiplet, aromatic and heterocyclic protons), 5.14–5.16 ppm (triplet, CH), 4.60 ppm (doublet, N–CH_2_), and 4.46 ppm (singlet, Ar–CH_2_), consistent with the ISN structure. M-β-CD (Figure 7b) showed typical signals corresponding to its glucopyranose units, with particular focus on H-3 and H-5 protons located inside the hydrophobic cavity.

Following complexation, noticeable chemical shift changes were observed in the H-3 and H-5 protons of M-β-CD, as shown in Table 1. For the freeze-dried (FD) complex, Δδ values were +0.086 ppm for H-3 and +0.026 ppm for H-5, while for the spray-dried (SD) complex, the shifts were −0.001 ppm for H-3 and −0.006 ppm for H-5. These changes confirmed molecular interactions between ISN and M-β-CD.

The chemical shift difference pattern observed (ΔδH-3 < ΔδH-5) is indicative of the complete inclusion of the ISN molecule within the cyclodextrin cavity [40]. Moreover, the proximity of the H-3 proton to the narrow rim of the M-β-CD cavity further suggests that ISN is oriented within the central hydrophobic region of the host molecule.

## 3. Materials and Methods

### 3.1. Materials

Isoconazole nitrate (ISN) was obtained from Deva Pharma, Istanbul, Türkiye, while methyl-beta-cyclodextrin (M-β-CD, average degree of substitution (DS) ≈ 0.7; corresponding molecular weight (MW) ≈ 1310 Da, based on a β-cyclodextrin backbone of 1135 Da) was purchased from Sigma-Aldrich, Darmstadt, Germany. Methanol (≥99.8%) and ethanol (≥95.0%) were supplied by Sigma-Aldrich, Darmstadt, Germany, whereas deuterated dimethyl sulfoxide (DMSO-d_6_, ≥99.9%) was also sourced from the same supplier. Potassium dihydrogen phosphate (KH_2_PO_4_, ≥99.0%) was obtained from J.T. Baker, Radnor, USA. Deionized water was prepared using a Milli-Q system from Millipore, Darmstadt, Germany, and pure water was used throughout all experiments. All solvents and reagents were of analytical grade and were used without further purification.

### 3.2. High-Performance Liquid Chromatography (HPLC) Analysis of Isoconazole Nitrate

The quantification of ISN and the evaluation of phase solubility studies were conducted using high-performance liquid chromatography (HPLC) in accordance with the United States Pharmacopeia (USP 31) monograph method [21,41]. An isocratic HPLC method was utilized, employing a Shimadzu LC-20A system (Shimadzu Corporation, Kyoto, Japan). The system was equipped with a pump, degasser, autosampler, column heater, and ultraviolet (UV) detector. Chromatographic separation was performed on a VDSpher 100 C18-E reversed-phase column (250 × 4.6 mm, 5 µm particle size), supplied by VDS Optilab, Berlin, Germany. The mobile phase consisted of a 50:50 (*v*/*v*) mixture of methanol and 0.05 M potassium dihydrogen phosphate buffer, with a constant flow rate of 0.5 mL/min. Detection was carried out at a wavelength of 210 nm, while the column temperature was maintained at 40 °C.

The method was validated over a concentration range of 20–500 µg/mL, ensuring the reliable quantification of ISN in all analyzed samples. The selection of 40 °C as the column temperature was based on its role in enhancing chromatographic efficiency by reducing mobile phase viscosity, thereby facilitating better mass transfer and sharper peak resolution. Moreover, operating at an elevated column temperature contributes to reduced peak broadening, more consistent retention times, and improved reproducibility of the chromatographic method [42].

### 3.3. Phase Solubility Studies

M-β-CD aqueous solutions were prepared at concentrations ranging from 2 × 10^−3^ M to 20 × 10^−3^ M. To each solution, an excess amount of ISN (20 mg) was added, followed by the addition of ethanol in a 3:1 (*v*/*v*) ratio to facilitate drug dispersion. The mixtures were continuously agitated using a horizontal shaker (WiseShake SHR-1D, Daihan Scientific, Seoul, Republic of Korea) at 25 °C for 24 h to reach equilibrium conditions.

After the incubation period, the samples were filtered using nylon syringe filters (0.45 µm pore size) to eliminate undissolved ISN. The filtrates were then subjected to HPLC analysis to quantify the dissolved ISN concentration. All experiments were performed in triplicate for accuracy and reproducibility. The obtained data were used to generate a phase solubility diagram, and the solubility profile was interpreted according to the Higuchi and Connors model [25].

### 3.4. Preparation of Isoconazole Nitrate/Methyl-β-Cyclodextrin Inclusion Complexes

#### 3.4.1. Physical Mixing Method

Accurately weighed quantities of isoconazole nitrate (ISN) and methyl-β-cyclodextrin (M-β-CD) were mixed in a 1:1 molar ratio. The components were thoroughly triturated using a mortar and pestle until a uniform and homogenous blend was achieved. This simple mixing approach aimed to ensure an even distribution of ISN throughout the M-β-CD matrix, although no molecular-level inclusion complexation was expected with this method.

#### 3.4.2. Spray-Drying Method

Precisely measured amounts of ISN and M-β-CD, in a 1:1 molar ratio, were dissolved separately in methanol and pure water, respectively. The two solutions were then combined and subjected to continuous stirring using a magnetic stirrer until a clear, homogeneous solution was obtained. The resulting solution was subsequently dried using a Büchi Nano Spray-Dryer B-90 (Büchi, Flawil, Switzerland), equipped with a 0.7 mm spray head. The spray-drying process was carried out at a feed temperature of 100 °C, an outlet temperature of 60 °C, and a feed rate of 7 mL/min. Following the conclusion of the drying process, the resulting fine powder was collected and stored for the purpose of further characterization [28].

#### 3.4.3. Lyophilization (Freeze-Drying) Method

Precisely measured amounts of ISN and M-β-CD, in a 1:1 molar ratio, were dissolved separately in methanol and pure water, respectively. The two prepared solutions were mixed and stirred continuously using a magnetic stirrer until a clear and uniform solution was obtained. This mixture was then rapidly frozen at −86 °C and subsequently freeze-dried using a Lyovac GT-2 freeze-dryer (Leybold-Heraeus, Cologne, Germany). The lyophilization process was conducted over approximately 48 h to ensure the complete removal of solvents and the formation of a stable, solid inclusion complex. The resulting dry powders were collected and stored at room temperature for further characterization.

### 3.5. Characterization of Inclusion Complexes

A detailed characterization of the ISN/M-β-CD inclusion complexes was performed to assess their physicochemical attributes and molecular interactions. The evaluation involved determining the drug content, analyzing aqueous solubility, and examining surface morphology and thermal behavior through DSC. Furthermore, structural insights and host–guest interactions were explored using ^1^H-NMR and FT-IR.

#### 3.5.1. Morphological Analysis via Scanning Electron Microscopy (SEM)

The surface morphology of ISN/M-β-CD inclusion complexes obtained by different preparation techniques was analyzed using scanning electron microscopy (SEM). A Zeiss SupraTM 50 VP SEM instrument (Carl Zeiss AG, Oberkochen, Germany) was used to evaluate particle shape, surface characteristics, and morphological alterations resulting from the complexation process.

#### 3.5.2. Determination of Isoconazole Nitrate Content in Inclusion Complexes

The amount of ISN incorporated into the M-β-CD inclusion complexes was determined by dissolving 1 mg of the complex in 1 mL of 50% methanol. The solution was then suitably diluted with the same solvent and analyzed by HPLC to quantify the ISN content within the complex.

#### 3.5.3. Solubility Analysis of Inclusion Complexes in Distilled Water

The solubility of ISN/M-β-CD inclusion complexes in distilled water was evaluated by adding an excess amount of the complexes to 1 mL of distilled water, allowing the formation of saturated solutions. These dispersions were subjected to continuous agitation at 25 °C for 24 h, using a horizontal shaker to achieve equilibrium. Following equilibration, the solutions were filtered using a 0.45 μm nylon filter (Minisart, Sartorius, Göttingen, Germany) and analyzed using HPLC to determine the solubility of ISN in the complexed form. The experiment was conducted in triplicate to ensure the reliability and reproducibility of the results.

#### 3.5.4. Thermal Analysis via Differential Scanning Calorimetry (DSC)

The thermal behavior of the ISN/M-β-CD inclusion complexes was analyzed using DSC. Approximately 4 mg of the sample was placed in a sealed aluminum crucible and subjected to controlled heating at a rate of 10 °C/min over a temperature range of 50–300 °C using a Shimadzu DSC-60 system (Shimadzu Corporation, Kyoto, Japan).

#### 3.5.5. Structural Analysis via Fourier-Transform Infrared Spectroscopy (FT-IR)

FT-IR analysis was performed using a Shimadzu FTIR-8400S spectrometer (Shimadzu Corporation, Kyoto, Japan) equipped with a ZnSe ATR crystal to investigate functional groups and molecular interactions in ISN/M-β-CD inclusion complexes. Spectra were recorded within the 4000–500 cm^−1^ range. Baseline correction and atmospheric compensation were applied using the Shimadzu IRsolution software (version 1.30) to improve spectral clarity.

#### 3.5.6. Molecular Interaction Analysis via Proton Nuclear Magnetic Resonance (¹H-NMR)

^1^H-NMR spectroscopy was employed to investigate the dynamic behavior and molecular interactions within the ISN/M-β-CD inclusion complexes. Due to chemical shifts induced by host–guest interactions, ^1^H-NMR spectroscopy provided valuable insight into complexation mechanisms. The ^1^H-NMR spectra of the ISN/M-β-CD inclusion complexes were obtained using a Bruker NMR spectrometer (Bruker Corporation, Billerica, MA, USA), with deuterated dimethyl sulfoxide (DMSO-d^6^) as the solvent. This methodological approach facilitated the precise evaluation of chemical shift changes associated with complex formation.

## 4. Conclusions

This study successfully demonstrated the formation of solid-state inclusion complexes of isoconazole nitrate (ISN) with methyl-β-cyclodextrin (M-β-CD) using spray-drying (SD) and freeze-drying (FD) techniques. Characterization results from ¹H-NMR, FT-IR, DSC, and SEM analyses confirmed the effective molecular interaction and encapsulation of ISN within the cyclodextrin cavity. Phase solubility studies showed that the complexes followed an AL-type profile with 1:1 stoichiometry, and the SD method significantly enhanced aqueous solubility, reaching approximately seven times that of ISN. Additionally, the SD-prepared complexes exhibited slightly higher drug content compared to FD complexes. These results underscore the suitability of spray-dried ISN/M-β-CD inclusion complexes as a promising formulation strategy to improve the solubility, stability, and delivery potential of ISN in pharmaceutical applications.

## Figures and Tables

**Table 1:** Chemical shifts (δ, ppm) and chemical shift differences (Δδ) of H-3 and H-5 protons of M-β-CD in free and ISN-complexed forms.

Protons	M-β-CD (Free) (δ, ppm)	SD Complex (δ, ppm)	Δδ (SD)	FD Complex (δ, ppm)	Δδ (FD)
H-3	3.461	3.460	−0.001	3.547	+0.086
H-5	3.347	3.341	−0.006	3.373	+0.026

Δδ = δ (complexed) − δ (free).

## Data Availability

The data presented in this study are available in this article.

## Abbreviations

ISN	Isoconazole nitrate
M-β-CD	Methyl-β-cyclodextrin
SEM	Scanning electron microscopy
FT-IR	Fourier-transform infrared spectroscopy
^1^H-NMR	Proton nuclear magnetic resonance
DSC	Differential scanning calorimetry
FD	Freeze-drying
SD	Spray-drying
HPLC	High-performance liquid chromatography
PM	Physical mixture
DMSO-d_6_	Deuterated dimethyl sulfoxide
DC%	Drug content percentage
RSD	Relative standard deviation
AL-type	Linear phase solubility diagram type A
K1:1	Stability constant for 1:1 drug–CD complex
*K_S_*	Stability constant
R²	Correlation coefficient
δ	Chemical shift (for NMR spectroscopy)
ppm	Parts per million
